# Review of Advancements in Managing Cardiogenic Shock: From Emergency Care Protocols to Long-Term Therapeutic Strategies

**DOI:** 10.3390/jcm13164841

**Published:** 2024-08-16

**Authors:** Amaia Martínez León, Pablo Bazal Chacón, Lorena Herrador Galindo, Julene Ugarriza Ortueta, María Plaza Martín, Pablo Pastor Pueyo, Gonzalo Luis Alonso Salinas

**Affiliations:** 1 Cardiology Department, Hospital Universitario de Navarra (HUN-NOU), Calle de Irunlarrea, 3, 31008 Pamplona, Spain; amaiamtzleon@gmail.com (A.M.L.); bazalpablo@gmail.com (P.B.C.); juleneugarriza@gmail.com (J.U.O.); 2 Navarrabiomed (Miguel Servet Foundation), Instituto de Investigación Sanitaria de Navarra (IdiSNA), 31008 Pamplona, Spain; 3 Heath Sciences Department, Universidad Pública de Navarra (UPNA-NUP), 31006 Pamplona, Spain; 4 Advanced Heart Failure and Cardiology Department, Hospital Universitario de Bellvitge, Carrer de la Feixa Llarga s/n, 08907 L’Hospitalet de Llobregat, Spain; lherradorgalindo@gmail.com; 5 Cardiology Department, Hospital Clínico Universitario de Valladolid, Av Ramón y Cajal 3, 47003 Valladolid, Spain; plazamaria20@gmail.com; 6 Cardiology Department, Hospital Universitari Arnau de Vilanova, Av Alcalde Rovira Roure, 80, 25198 Lleida, Spain; ppastor.lleida.ics@gencat.cat

**Keywords:** cardiogenic shock, mechanical circulatory support, acute myocardial infarction, shock team, cardiac critical care, advanced heart failure

## Abstract

Cardiogenic shock (CS) is a complex multifactorial clinical syndrome of end-organ hypoperfusion that could be associated with multisystem organ failure, presenting a diverse range of causes and symptoms. Despite improving survival in recent years due to new advancements, CS still carries a high risk of severe morbidity and mortality. Recent research has focused on improving early detection and understanding of CS through standardized team approaches, detailed hemodynamic assessment, and selective use of temporary mechanical circulatory support devices, leading to better patient outcomes. This review examines CS pathophysiology, emerging classifications, current drug and device therapies, standardized team management strategies, and regionalized care systems aimed at optimizing shock outcomes. Furthermore, we identify gaps in knowledge and outline future research needs.

## 1. Introduction

Cardiogenic shock (CS) is a life-threatening syndrome resulting from severe impairment of ventricular pump function. This leads to systemic hypoperfusion and persistently high mortality rates despite establishing targeted treatment [1].

Cardiogenic shock (CS) is characterized by sustained hypotension (SBP < 90 mmHg or vasopressor requirement) and tissue hypoperfusion secondary to reduced cardiac output (CI < 2.2 L/min/m^2^) and congestion (PCWP > 15 mmHg, or pulmonary congestion on imaging, or CVP > 12 mmHg). While this “cold and wet” phenotype is the most common, not all patients with CS present with these exact hemodynamic parameters. Despite phenotypic variability, a low cardiac index is universally observed in CS. However, ventricular preload, volume status, and systemic vascular resistance can vary considerably among patients [2] (Table 1).

Euvolemic cardiogenic shock (CS), often termed “cold and dry”, typically presents in patients with chronic heart failure experiencing a subacute decompensation. These patients usually respond well to diuretics and have lower pulmonary capillary wedge pressures compared to those with classic CS.

In contrast, “wet and warm” CS is commonly seen following myocardial infarction, characterized by a systemic inflammatory response, decreased systemic vascular resistance, and an increased risk of sepsis and mortality [1].

Despite comparable cardiac function, normotensive patients with cardiogenic shock demonstrate elevated systemic vascular resistance, indicating a risk of tissue hypoperfusion. This finding highlights the importance of assessing systemic vascular resistance in patients with cardiogenic shock, even in the absence of overt hypotension [1,2].

## 2. Pathophysiology

CS may develop acutely or subacutely, and it can be precipitated by many etiologies, such as AMI, heart failure (HF) cases complicated by CS, acute myocarditis, valvular dysfunction, right ventricle shock, or postcardiotomy shock. AMI is the archetypal model used to understand the pathophysiology of CS [2].

Progressive hemodynamic deterioration leading to CS results from a critical reduction in contractile mass due to ischemic or necrotic myocardium. The resulting decrease in cardiac output lowers arterial blood pressure, reduces coronary perfusion pressure, and increases left ventricular end-diastolic pressure. This vicious cycle exacerbates myocardial ischemia, further impairing left ventricular function and cardiac output.

To compensate for the reduced cardiac output, a series of neurohumoral responses is activated. Sympathetic activation increases heart rate, myocardial contractility, and systemic vascular resistance, redirecting blood flow to vital organs. Renin–angiotensin–aldosterone system activation and antidiuretic hormone release promote fluid retention and vasoconstriction.

**Figure 1:**
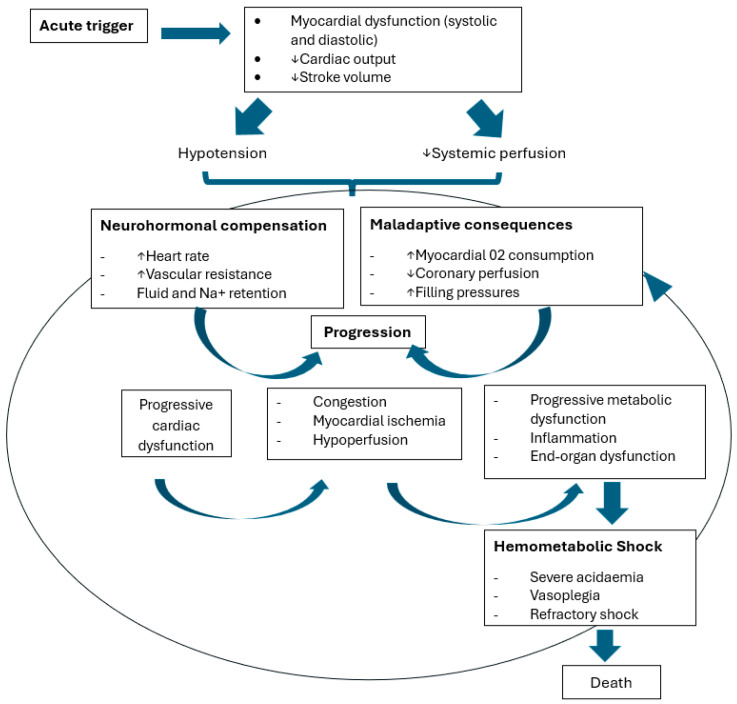
Pathophysiology of cardiogenic shock. Adapted from [1,2]. An abrupt drop in myocardial contractility produces myocardial dysfunction and a reduction in stroke volume. This leads to hypotension and decreased peripheral perfusion, triggering a reflex increase in cardiac and systemic vascular resistance, propagating myocardial ischemia. A vicious cycle of hypoperfusion, end-organ dysfunction, and inflammation occurs, ultimately leading to refractory hemometabolic shock and death.

However, these compensatory mechanisms eventually lead to decompensation. Tissue hypoxia, acidosis, and depletion of ATP stores impair cellular function. Loss of vascular endothelial integrity and microvascular thrombosis contribute to multiorgan failure. Systemic inflammation and impaired nitric oxide production further exacerbate myocardial dysfunction and vasoconstriction. Ultimately, this downward spiral results in decreased tissue perfusion, lactic acidosis, and death (Figure 1) [1,2,3].

## 3. Epidemiology and Prognosis

A decade ago, 80% of CS cases were associated with AMI, with an estimated prevalence of around 5%. However, evolving definitions and advancements in acute care and early reperfusion in AMI have significantly decreased its incidence [4,5].

Presently, other causes of CS are increasing. According to a U.S. registry with over 140,000 patients with CS from various etiologies, the proportion related to AMI dropped from 65.3% to 45.6% between 2005 and 2014 [6]. Furthermore, in a contemporary dataset from intensive care units (ICUs) in the U.S. and Canada, only one-third of CS cases were related to AMI, while the remainder included ischemic cardiomyopathy without acute coronary syndromes (18%), non-ischemic cardiomyopathy (28%), and other causes (e.g., ventricular arrythmias or valvular heart disease) in 17% [7].

CS remains prevalent, accounting for 2–5% of acute HF presentations and 14–16% in ICUs [6,8,9]. Despite advancements, the hospital mortality rate for CS remains elevated, ranging from 30 to 60%, with almost half of the deaths occurring within the first 24 h. The one-year mortality rate stands at 50–60%, with a peak in incidence during the first 30–60 days after diagnosis [4,10,11].

## 4. Clinical Presentation, Early Detection, Phenotyping, Monitoring, and Risk Stratification

Immediate assessment of hypoperfusion signs and continuous monitoring of SBP, rhythm, respiratory rate, and saturation are recommended. In addition, pulse pressure should be closely monitored, especially in patients with normotensive CS. A SBP ≥ 90 mmHg or mean arterial pressure (MAP) in the range of 60–65 mmHg is generally recommended, but this target has not been validated in randomized clinical trials (RCTs) [4]. Recent studies have found an association between higher MAP levels (at least 70 mmHg) and better prognosis in CS [12,13].

An electrocardiogram should be obtained and interpreted immediately in any patient with hemodynamic compromise with suspected cardiac origin to exclude bradi/tachyarrhythmia and assess repolarization abnormalities indicating acute myocardial ischemia or inflammation, as well as other signs of cardiomyopathy or pericardial disease.

Bedside-focused echocardiography (BSFE) should be performed as soon as possible when CS is suspected to obtain critical diagnostic and prognostic information (Table 2).

Chest X-ray remains important for the evaluation of congestion and to monitor the catheter and cardiac device position [14].

The insertion of a central venous catheter is recommended in all patients with CS to allow the transduction of CVP and access for vasoactive drug administration. Urine output also needs to be monitored hourly through catheter drainage. An arterial catheter allows for constant monitoring of the blood pressure [4]. Arterial blood gas analysis should be performed when a precise measurement of O_2_ and CO_2_ partial pressure is needed (i.e., patients with respiratory distress). Lactate and pH levels should be measured in patients with cardiogenic shock [14].

Hemodynamic monitoring with pulmonary artery catheters (PACs) has been associated with better outcomes in contemporary CS observational studies [15,16]. Thus, invasive monitoring is advisable to confirm the hemodynamic phenotype, ascertain left and right filling pressures, complete prognostic assessment, and guide therapeutic strategies. Specifically, the pulmonary artery pulsatility index (PAPi) and the Cardiac Power Output (CPO) are recommended for prognostic purposes [17,18]. However, to date, PAC-guided management has not been prospectively tested in patients with CS, although previous RCTs have failed to show a survival advantage in acute decompensated HF or ICU patients. Currently, recruitment is ongoing for the PACCS trial, a multicenter, randomized study designed to assess whether early invasive hemodynamic assessment and ongoing management with a PAC in patients with CS due to acutely decompensated HF is associated with a lower risk of in-hospital mortality [19].

In recent years, non-invasive or minimally invasive devices based on pulse–waveform analysis have been developed and might represent an alternative for patients in earlier shock stages or with contraindications for invasive monitoring [20].

The early revascularization strategy represents the cornerstone in the management of patients presenting with CS complicating ACS (AMICS) (see “Revascularization approach in AMICS”). In suspected non-AMICS, angiography could be performed during admission to rule out acute or chronic coronary artery disease.

### 4.1. Contemporary Risk Stratification of Cardiogenic Shock

Following classic definitions and the Killip and Kimball (KK) classification, AMICS has been traditionally considered a dichotomous diagnosis; patients were deemed to be hemodynamically stable or in a hypotension–hypoperfusion state. However, this approach lacks the capacity to depict the complexity and variety of hemodynamic scenarios of CS, precluding timely diagnosis of early-stage cases and the application of individualized treatment strategies.

To overcome these limitations and improve diagnostic and prognostic accuracy, in 2019, the Society of Cardiovascular Angiography and Intervention published a new model for the diagnosis and classification of CS, dividing this clinical situation into five stages with increasing severity [21]. At each stage, cardiac arrest is proposed as a disease modifier, considering its impact on both pathophysiology and clinical evolution. 

**Figure 2:**
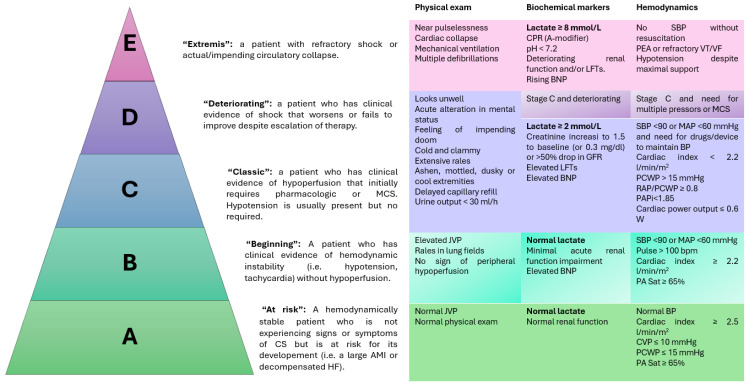
SCAI classification and definition of each stage. Adapted from [21,22]. BNP, B-type natriuretic peptide; CPR, cardiopulmonary resuscitation; CVP, central venous pressure; GFR, glomerular filtration rate; JVP, jugular venous pressure; LFT, liver function test; MAP, mean arterial pressure; MCS, mechanical circulatory support; PA, pulmonary artery; PCWP, pulmonary capillary wedge pressure; SBP, systolic blood pressure.

Recently, an update of this classification has validated the use of the SCAI classification as a prognostic tool and established more specific criteria for the definition of each stage, depending on the level of circulatory support required and the levels of lacticaemia (Figure 2) [22].

Furthermore, it should be noted that in the AMICS scenario, 50% of cases present overt CS at admission, while in the other half the hemodynamic deterioration occurs in the hospital, usually within the first 12–24 h [23]. Therefore, it is critical to detect patients at risk of hemodynamic worsening or with incipient signs of instability before the disease progresses to advanced stages with a worse prognosis.

### 4.2. Prediction and Early Detection of AMICS

Several tools are available to predict the development of CS in patients admitted for AMI. Given their simplicity and prognostic value, we consider “Shock Index” and “ORBI score” the most practical ones.

### 4.3. Shock Index (SI)

Defined as the ratio between the heart rate and systolic blood pressure, values below 0.7 are considered normal. It reflects the integrated response of the cardiovascular and nervous system in response to an acute disease. Increased values indicate a situation of organic stress and catecholaminergic discharge, which, however, is not sufficient to maintain adequate blood pressure.

Numerous studies have established the prognostic value of the SI in a variety of clinical scenarios, including acute coronary syndrome, consistently showing a proportional relationship between the SI value and the development of shock and mortality [24,25]. Within AMI, the “crossover” between the heart rate and systolic blood pressure (SI > 1) usually indicates a significant decline in stroke volume, physiologically compensated by an increasing heart rate to maintain cardiac output. Therefore, this finding can be interpreted as an alarm signal that reveals an unstable hemodynamic situation, with possible evolution towards overt CS if stabilizing measures are not implemented.

### 4.4. ORBI Score

This prognostic tool can be applied to ST-elevation AMI patients without shock on admission to predict the subsequent occurrence of shock using a set of clinical data available at the time of primary percutaneous angioplasty [26]. The score is obtained by entering a series of parameters in a computerized calculator available on the freely accessible website [27].

A score greater than 12 points implies a high probability of developing CS during admission, thus identifying a population of patients who, despite not meeting shock criteria at the time of primary angioplasty, are at risk of hemodynamic deterioration and consequently high in-hospital mortality.

**Figure 3:**
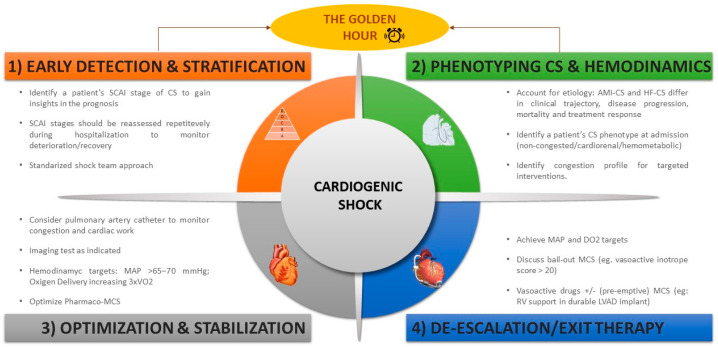
Global approach to CS. Early detection, differential diagnosis, and risk stratification. Adapted from [28]. AMI-CS, cardiogenic shock complicating acute myocardial infarction; CS, cardiogenic shock; DO_2_, oxygen delivery; HF-CS, heart-failure-related CS; LVAD, left ventricular assist device; MAP, mean arterial pressure; MCS, mechanical circulatory support; RV, right ventricle.

In summary, early detection of patients at risk of CS is crucial. This can be achieved by using a combination of clinical, hemodynamic, and echocardiographic data based on a standardized shock team approach. Hemodynamic monitoring is recommended to complete phenotyping, ascertain filling pressures, improve prognostic prediction, and guide therapeutic maneuvers. Hemodynamic support should be individualized depending on the patient’s characteristics, SCAI shock stage, and phenotype. Recovery of organ function and an “Exit Therapy” are essential for survival from CS. Therefore, the therapeutic goals in CS should be restoring tissue perfusion and organ function, facilitating recovery of cardiac function, or, in the absence of adequate recovery, bridging to an “Exit Therapy” (Figure 3). The “CS journey” can therefore be conceptualized into four phases: Recognize/Rescue–Optimization–Stabilization–de-Escalation/Exit Therapy (R-O-S-E) [28].

## 5. Stabilization and Resuscitation Strategy in Acute Setting

1. Medical–pharmacological management: Vasopressors and Inotropics

Vasopressor and inotropic drugs are the major initial interventions for reversing hypotension and improving vital organ perfusion. Failure to improve blood pressure with these agents is an important prognostic sign [3]. Despite a class IIb indication in current guidelines, these drugs are administered in approximately 90% of patients with CS in clinical practice, especially in non-ischemic etiology [29].

Catecholamines and phosphodiesterase-III (PDE-III) inhibitors all increase myocardial contractility through an increase in intracellular Ca^2+^ concentrations. For this reason, these agents were classified as cardiac calcitropes. Catecholamine infusions should be carefully titrated to obtain a balance between increasing the coronary perfusion pressure and increasing the oxygen demand so that myocardial ischemia is not exacerbated. Moreover, excessive peripheral vasoconstriction decreases tissue perfusion and increases afterload as well as filling pressures, and excessive tachycardia or arrhythmias can be stimulated [29].

Dobutamine is a synthetic catecholamine with predominantly β1-adrenergic effects (3:1 ratio compared to β2-stimulation). It is a potent inotrope with weak chronotropic activity. Due to its combined α1-adrenergic agonism and antagonism as well as β2-stimulation, the net vascular effect is often mild vasodilatation. Dobutamine increases cardiac output in patients with severe HF by increasing stroke volume and decreasing systemic vascular resistance. It is particularly effective in right ventricular shock because it also reduces right afterload [30].

Norepinephrine is a natural catecholamine with a strong peripheral α1-adrenergic effect and moderate β1-adrenergic effects that generates a potent venous and arterial vasoconstriction but a less potent inotropism. It may reduce cardiac output in patients with cardiac dysfunction because of an afterload increase.

Dopamine is an endogenous central neurotransmitter and a precursor of norepinephrine. Low doses (2–5 μg/kg per minute) increase stroke volume and renal perfusion by stimulating dopamine receptors. Intermediate doses have a dose-dependent β1-adrenergic receptor effect, increasing inotropy and chronotropy. High doses (15–20 μg/kg per min) activate α-adrenergic receptors, increasing vascular resistance [29]. There is limited evidence of the efficacy or dopamine from RCTs. The SOAP II RCT showed that dopamine was associated with an increased rate of death at 28 days and with more arrhythmic events among patients with CS compared to norepinephrine [31].

PDE-III inhibitors, such as milrinone, increase inotropy and produce a reduction in systemic vascular resistance. They also have lusitropic properties that lead to an improvement in diastolic function. They can be useful in low-output states when the patient is relatively stable by augmenting myocardial contractility and producing peripheral vasodilation [29]. The DOREMI trial compared dobutamine and milrinone as first-line inotropes in predominantly HF-CS, finding no difference between groups with respect to survival, efficacy, or safety [32]. Importantly, due to their pharmacodynamic characteristics, PDE-III inhibitors maintain their favorable hemodynamic effects in patients on ongoing β-blocker treatment [3,29].

Finally, Levosimendan is a calcium sensitizer that can increase cardiac inotropy through a direct effect on cardiac troponin C. The most-studied use of this drug is in the advanced HF context, but the experience in CS is limited [33,34].

Table 3 provides an overview of the primary indications and risks, along with the dosing for the key inotropic and vasopressor drugs commonly used in clinical practice.

2. Mechanical ventilation

Left ventricular dysfunction and acute kidney injury may lead to acute respiratory failure (ARF) in CS [35]. ARF causes an increase in oxygen consumption that results in a worsening low-cardiac output state and hypoperfusion [36]. Positive pressure ventilation (PPV) has several benefits in CS. It improves oxygen supply due to a decrease in alveolo-interstitial oedema and alveolar recruitment [37,38]. In addition, PPV diminishes oxygen consumption by reducing the work of breathing. Finally, PPV could increase cardiac output due to a reduction in preload and left ventricular afterload [36,39]. On the other hand, non-invasive PPV is crucial in pulmonary oedema, but its benefit in CS is not clear due to the absence of hemodynamic stability and frequent low levels of consciousness.

There is a lack of RCTs that compare non-invasive versus invasive PPV in CS. It seems reasonable to consider non-invasive PPV in the early stages of CS with close monitoring of the initial response. Invasive PPV should be the first choice rather than non-invasive PPV in profound CS and patients with neurologic impairment.

However, there are no specific recommendations about the invasive PPV mode in CS. The initial PEEP level is 5 cmH_2_O, and it should be gradually increased to the optimal value with an oxygen saturation target between 92 and 98% [40]. The tidal volume should be set from 6 to 8 mL/kg of the ideal body weight in order to avoid deleterious effects of hypercapnia on the right ventricle secondary to the huge increase in pulmonary arterial resistance [41]. Finally, it is important to consider the presence of severe right ventricular failure while proceeding to PPV in CS as these subgroups of patients are at high risk of hemodynamic deterioration [42].

3. Renal replacement therapy

There are no specific studies that evaluate the benefit of early renal replacement therapy (RRT) in CS. Generally, there is no evidence that supports early RRT in terms of improved survival in shock of different etiologies [43,44]. Continuous RRT is more commonly used for patients with CS than intermittent hemodialysis, as these patients often do not hemodynamically tolerate fluid shifts associated with intermittent hemodialysis.

It seems reasonable to initiate continuous RRT in CS only when there is refractory pulmonary congestion, persistent oliguria, or severe metabolic disturbances, such as hyperkalemia, metabolic acidosis, or uremia [42].

4. Mechanical circulatory support

Mechanical circulatory support (MCS) in CS should be considered with a IIa level of recommendation, as stated in European [45] and American [46] guidelines. Inotropes and vasopressors have a lower degree of recommendation in this context (IIb in European guidelines).

Although the class of recommendation is lower for vasoactive drugs, it seems reasonable to consider MCS only in patients who cannot be stabilized with vasoactive drugs (mainly SCAI D and E) [22]. Prompt implantation in selected patients [47], a standardized approach to CS and high-volume centers, may improve outcomes [48,49].

The device selection algorithm must take into account different aspects: the CS phenotype (left, right, or biventricular failure) and etiology (ischemic, non-ischemic, acute on chronic, etc.), the SCAI category, previous cardiac arrest in non-shockable rhythm, and concomitant ARF or electrical instability, among others. It is crucial to know specific contraindications of devices and to try to avoid futility. MCS should be discouraged if neurological impairment is presumed, there is irreversible end-organ failure, or a low chance of recovery when advanced heart failure therapies are not considered. Table 4 shows different devices and their specific characteristics.

### Mechanical Circulatory Support during Percutaneous Coronary Intervention in CS Complicating Acute Myocardial Infarction

Percutaneous MCS is an attractive tool to consider in CS patients undergoing percutaneous coronary intervention (PCI), with several potential benefits. First of all, left ventricular unloading prior to PCI in CS reduces filling pressures and myocardial oxygen consumption and has been shown to reduce reperfusion injury and infarct size [50,51,52,53,54]. Second, MCS increases cardiac power, reducing the hypotension and hypoperfusion vicious cycle, and it provides hemodynamic support during PCI [55]. And, third, MCS could help to reduce the dose of potentially harmful drugs, such as vasopressors and inotropes, that increase oxygen consumption, aggravating myocardial damage in AMICS patients [56].

The first MCS used was the intra-aortic balloon pump (IABP). Old studies suggested that the use of IABP might have a potential benefit, especially in AMI patients, as a result of afterload reduction and diastolic augmentation with improvement in coronary perfusion [57,58]. In fact, in the SHOCK trial, IABP was implanted in 86% of both groups [59]. However, in a small clinical trial, the use of IABP on top of medical care in patients with AMICS was only associated with lower levels of BNP, but no significant differences in APACHE II scoring, CI, and systemic inflammatory activation were found compared to standard care alone [60]. Moreover, in the IABP-SHOCK II trial, the use of IABP in AMICS where early revascularization was performed failed to provide a significant reduction in 30-day mortality compared with optimal medical treatment, irrespective of the moment of IABP insertion (before or after PCI), although the majority of them were implanted after revascularization. Interestingly, no significant improvement in surrogate endpoints, such as blood pressure, heart rate, serum lactate, and C-reactive protein, was found in the intervention group [61]. The absence of a significant benefit in terms of mortality was confirmed at the 1- and 6-year follow-ups [62,63]. Thus, the supposed hemodynamic benefit could be modest enough to preclude a significant improvement in clinical outcomes in AMICS [64]. Nowadays, routine use of IABP in this context is not recommended during PCI [65]. Nevertheless, IABP might be useful in selected populations in CS, in particular in those presenting with a mechanical complication, such as a ventricular septal defect or acute mitral regurgitation [66,67,68].

Given the unfavorable results of the IABP-SHOCK II, the use of IABP has been decreased in recent years, increasing the use of alternative MCS devices, such as Impella^®^ (Abiomed, Danvers, MA, USA), especially in patients undergoing PCI [69,70]. The hemodynamic effect generated by the microaxial flow pump is much greater than with IABP in terms of increased cardiac output and ventricular unloading. Furthermore, it does not require synchronization, it works independently of the LV function, and its sheath allows for a single access site during PCI, thus simplifying and reducing the procedure time and fluoroscopy [55,71,72]. Despite its attractive hemodynamic profile, the cost of the device, a non-negligible rate of complications, especially major bleeding related to the large bore access site (28%) and hemolysis (2%), and the negative results in registries and small clinical trials restricted the recommendations of the use of the Impella^®^ device to selected patients as a bridge to decision during the last years [70,73,74,75,76].

However, a recently published study, the DANGER SHOCK trial, was the first clinical trial to probe a survival benefit with MCS in CS patients. A total of 360 patients with STEMI in CS were randomized to a combination of Impella^®^ and standard care or standard care alone, including emergent revascularization [77]. The primary end-point, all-cause death at 180 days, was significantly lower in the Impella^®^ group with a number needed to treat of eight patients [78]. Despite the survival benefit, concerns have been raised regarding safety and external validation of these results. Even though the trial was performed in experienced centers, mortality in the control group was strikingly high. Furthermore, the time of inclusion was of 10 years, which could indirectly reflect high patient selection. On the other hand, the rate of complications (RRT, major bleeding and limb ischemia) was higher in the intervention group [79,80].

Evidence regarding the time of MCS initiation, before or after PCI, is scarce. However, available data suggest that whenever Impella^®^ is planned to be inserted, pre-PCI implantation seems to be the most favorable strategy, with a potential survival benefit [81,82,83]. Remarkably, nearly 85% of patients in the DANGER-SHOCK trial received Impella^®^ prior to PCI in the intervention group.

The third potential percutaneous MCS available in the context of PCI in AMICS is extracorporeal membrane oxygenation (VA-ECMO). This MCS may be suitable in CS patients with biventricular dysfunction and/or advances stages of CS. However, to date, there is no clear evidence of a survival benefit with ECMO implantation in CS [84,85]. Two recent trials assessed the survival effect with VA-ECMO: the EUROSHOCK trial, which was stopped prematurely due to a slow recruitment rate, and the ECLS-SHOCK trial, where 420 patients were randomized to ECMO and medical treatment vs. medical treatment alone. In this trial, no significant differences in mortality at 30 days were found between groups.

Similarly to what is described for the rest of the percutaneous MCSs, the implantation of VA-ECMO prior to revascularization is related to better clinical outcomes [83,86]. However, once again, data came from observational or retrospective studies, and the ECLS-SHOCK trial protocol did not require implantation prior to revascularization [87]. In fact, in this trial, ECMO was initiated after revascularization in 52% of the intervention group, and nearly 15% of the control group received another MCS, with Impella CP^®^ being the most widely used (85%) [88,89,90].

## 6. Revascularization Approach in Cardiogenic Shock Complicating Acute Myocardial Infarction

Coronary angiography continues to be a cornerstone in the diagnostic pathway in patients presenting with CS [91]. As the main cause of CS, acute coronary syndrome must be ruled out unless another potential cause is previously diagnosed [5,65,92]. This is even more decisive if ongoing ischemia is suspected, where emergent coronary angiography and early revascularization have been shown to improve survival [93].

In the SHOCK trial, a total of 302 patients were randomized to early revascularization or medical stabilization alone. Although no significant differences were found at 30 days, at the 6-month follow-up, revascularization was associated with a significant reduction in mortality compared to medical treatment [59].

Indeed, in the AMI registry carried out in 83 hospitals in Switzerland, emergent revascularization in the context of a primary PCI program was the main independent factor related to a decrease of nearly 50% of in-hospital mortality despite an increase in CS at presentation, from 2.5% to 4.6%, from 1997 to 2017 [94].

Single-vessel disease is an infrequent finding in coronary angiography in CS patients. Furthermore, multivessel disease is present in approximately 80% of patients with AMICS [93,95]. Management of non-culprit lesions has been a matter of controversy over the last years. Initially, complete revascularization during the index procedure was encouraged with the supposition that non-culprit significant lesions in the context of hypotension could lead to a worsening in cardiac output driven by ischemia over a large territory and would aggravate the hypoperfusion status [96,97,98,99]. However, observational data from big registries and a metanalysis did not support this hypothesis and suggested a potential harmful effect of immediate multivessel revascularization [94,100,101]. Finally, in 2017, the CULPRIT SHOCK trial was published [102]. In that study, 706 patients with MI in CS and multivessel disease were randomized to receive immediate culprit-only or multivessel revascularization [103]. Culprit-only revascularization was associated with a significant reduction of the primary endpoint, a combination of all-cause mortality and severe renal dysfunction [102]. At the one-year follow-up, differences in the primary endpoint did not change; however, there were no significant differences in mortality alone, and the rates of heart failure rehospitalization and repeated revascularization were higher in the culprit-only revascularization group [93]. This results could indicate that a staged revascularization in multivessel disease in CS may have a potential benefit [69,104]. However, specific data are scarce in this field, as CS patients are usually excluded or underrepresented in trials (i.e., in the COMPLETE trial, only 10% of the patients included were at ≥Killip 2 at presentation) [105]. With respect to the available evidence, current guidelines recommend exclusive revascularization of the culprit vessel during the index procedure [65].

## 7. Long-Term Therapies: Heart Transplantation and Durable Mechanical Circulatory Support

Exit strategies must be planned from the initial phases of CS [28]. While managing CS, achieving hemodynamic stability and restoring both end-organ and metabolic function are prerequisites for considering temporary MCS weaning. However, registry data show that only around 35% of patients survive with their native heart [106]. For this reason, simultaneous evaluation of the need and eligibility for heart replacement therapies (HRTs) is crucial, guiding the up-titration of circulatory support and helping in the selection of the appropriate MCS option [107,108].

The decision-making process requires a correct understanding of the patient’s specific HF phenotype and etiology [109]. For instance, cardiac recovery is more likely in postpartum cardiomyopathy or chemotherapy-induced dilated cardiomyopathy than in ischemic cardiomyopathy [110]. Consequently, the entire CS team has to be involved, including advanced HF specialists, interventional cardiology, cardiac surgeons, and critical care specialists. Non-transplant centers are encouraged to contact a transplant center in order to discuss the potential heart transplant (HTx) candidacy [111].

Currently, 40–50% of HTx patients are transplanted while on MCS. For patients bridged to transplant under temporary MCS, isolated left ventricle support has shown better outcomes compared to biventricular assist devices and ECMO [112]. Additionally, the introduction of high-capacity Impella^®^ pumps has increased the use of axial flow pumps, showing high success rates for weaning or bridging to HRT while allowing patient rehabilitation [113].

The advancements in left ventricular assist device (LVAD) technology, with improved hemocompatibility and reduced bleeding risks of HeartMate 3^®^ (Abbot, Santa Clara, CA, USA) [114,115], have established LVADs as a viable long-term solution for patients ineligible for HTx as a destination therapy (DT). Moreover, there is growing interest in the potential for myocardial recovery in patients with LVADs due to ventricular unloading, and it should be pursued and evaluated, especially in those patients with specific etiologies known for better recovery chances and shorter HF duration [116,117]. In the setting of bridge to transplantation (BTT) therapy, LVADs have been associated with a higher risk of primary graft dysfunction but have not demonstrated higher mortality rates after HTx [118].

Eligibility for LVAD (both as DT or BTT) or HTx should be evaluated concurrently. Factors to consider include age, etiology and duration of HF, right heart function, potential for myocardial recovery, allosensitization, irreversible pulmonary hypertension, frailty, etc. When both strategies are suitable, clinicians must balance short-term risks of tMCS with long-term risks of LVAD support, considering expected waiting times for HTx (depending on blood type, patient size, country donation rates, etc.) and taking into account that stable LVAD patients are not eligible for urgent status [119]. Table 5 summarizes patient selection criteria for both techniques.

Regarding the prognosis, the current 2-year survival rates for patients receiving continuous-flow ventricular assist devices are comparable to those of transplant recipients, although adverse events negatively impact quality of life. The actuarial survival was 90% at 1 year and 85% at 2 years (with approximately 80% free from disabling stroke), while the 2-year survival for transplant patients is around 80–90% [114]. In the long term, transplant survival remains unmatched, with a median life expectancy of approximately 12.5 years [45].

Changes in the United Network for Organ Sharing (UNOS) HTx allocation policy have led to a reduction in LVAD implantation and a rise in temporary MCS [120] in the US, with no differences in post-transplant mortality rates. Conversely, in Europe, BTT LVAD use has increased within the last years. This trend suggests regional variation influenced by differing allocation policies and donor availability [119].

Despite the numerous exit strategy options, it is crucial to plan the patient’s roadmap from the initial stages of CS to select the optimal temporary MCS. This approach aims for a successful exit strategy (recovery or HRT) with minimal morbidity, as exposure to multiple MCSs has been shown to increase morbidity and mortality [106,113].

Finally, involvement of palliative care professionals is recommended to address end-of-life concerns and withdrawal of care with patients and families when exit strategies are not viable [107].

## 8. Regional Systems for the Management of CS

### 8.1. Shock Teams

**Figure 4:**
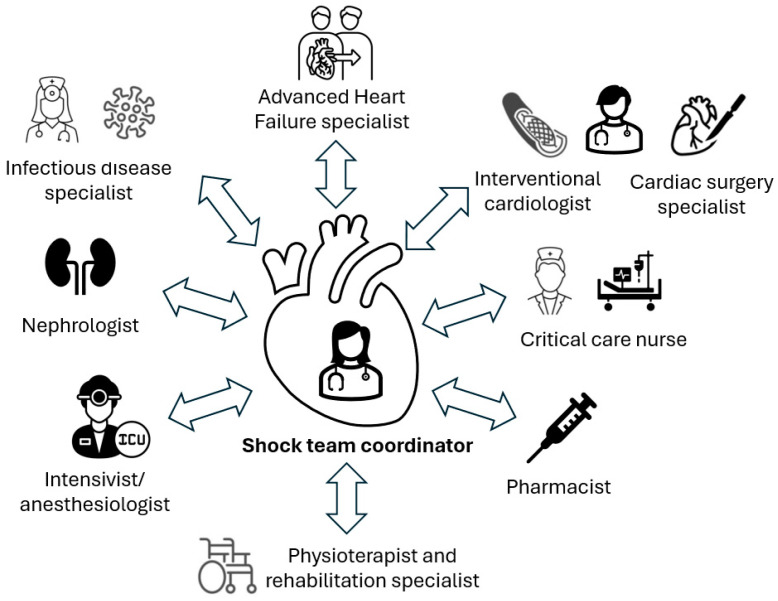
Shock team multidisciplinary approach.

Given the complexity and multiorgan derangement that often accompany CS, a multidisciplinary approach is a key element to achieving good clinical results [121]. The combined work of cardiologists, intensivists, anesthesiologists, infectious disease specialists, nephrologists, critical care nurses, and physiotherapists, among others, is necessary for the optimal care of this complex clinical scenario. Both cardiologists with expertise in critical cardiac care or in advanced HF, as well as intensivists or anesthesiologists with comprehensive cardiac training, are usually shock team leaders, with variations depending on the local organization (Figure 4).

### 8.2. Regional Networks for the Management of CS

Patients with CS may present in medical facilities with varying degrees of complexity and capabilities, as well as in the pre-hospital environment. This heterogeneity might impact clinical results and hinder equality, as the management of CS in high-volume hospitals with specific cardiac ICUs is associated with better prognosis [122]. With the aim of establishing a homogeneous approach between different centers, providing best-quality care, and mitigating inequality, various models of regional networks for the management of CS have been proposed [123,124]. The current consensus is to develop a pyramidal organization consisting of three levels, connected through a “hub and spoke” model.

Level 3 centers (small community hospitals) might represent the first medical contact, and they have an important role in achieving early diagnosis and performing the first stabilization measures. Level 2 (advanced) centers are characterized by the presence of cardiac catheterization laboratory and the capacity to perform 24/7 primary PCI, as well as providing temporary MCS and hemodynamic monitoring. Finally, level 1 (referral) centers are defined as those with multidisciplinary shock teams with extensive experience and competence in critical cardiac care; they are equipped with all types of MCS systems and can provide long-term care with heart transplantation or LVAD.

**Figure 5:**
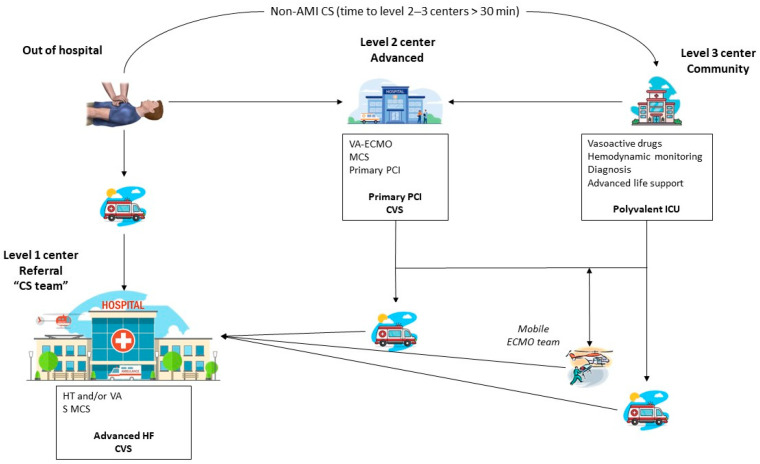
Organization of regional cardiogenic shock networks. Adapted from [123]. AMI, acute myocardial infarction; CS, cardiogenic shock; CVS, cardiovascular surgery; HF, heart failure; HT, heart transplant; ICU, intensive care unit; MCS, mechanical circulatory support; PCI, percutaneous coronary intervention; S, surgery; VA, mid/long-term ventricular assistance; VA-ECMO, venoarterial extracorporeal membrane oxygenation.

Those three types of hospitals must be coordinated and work in close collaboration. Communication between different centers must be direct and fluid to avoid delays in reperfusion or hemodynamic support interventions. Besides providing telematic assistance, referral hospitals should ensure the capability to implement MCS in level 2 and 3 centers if the patient´s clinical condition is too unstable for non-supported transfer through the deployment of mobile ECMO teams available 24/7 (Figure 5) [123].

### 8.3. Mobile ECMO

#### 8.3.1. Out-of-Hospital Extracorporeal Cardio-Pulmonary Resuscitation

Extracorporeal cardiopulmonary resuscitation (ECPR) may be considered (IIb level of recommendation) in selected patients when conventional cardio-pulmonary resuscitation is failing—including in the pre-hospital setting if feasible [125,126]. ECPR should be restricted to young patients (below 65 years old) without comorbidities, shockable rhythms, and a presumably reversible CA origin. Pre-hospital mobile ECPR programs are scarce, but theoretically they could decrease the time to ECMO circulation restoration [127] and might be cost-effective [128] in out-of-hospital CA.

#### 8.3.2. Primary VA-ECMO Transport

Patients with refractory CS admitted to remote hospitals without MCS are exposed to high risk of death. Mobile-ECMO teams provide access to VA-ECMO and transfer the patient to high-volume centers. There are multiple registries that describe similar survival rates between primary VA-ECMO transport cohort and in-hospital VA-ECMO implantation group [129,130].

## 9. Current and Future Perspectives

Despite advances in pharmacological treatments, monitoring, revascularization, and MCS, CS remains the most common cause of in-hospital death in patients with AMI. While gaps in evidence are extensive and data from RCTs are limited due to inherent recruitment challenges, this field presents several promising avenues for future research.

For instance, developing novel biomarkers for early detection and risk stratification could revolutionize patient management. Furthermore, investigating the efficacy of emerging therapeutic strategies, such as novel pharmacological agents or combination therapies, in conjunction with optimized mechanical circulatory support, warrants further exploration. Additionally, the establishment of large-scale, multicenter registries could facilitate the identification of high-risk patient subgroups and inform the design of future clinical trials.

The standardization of care for CS led by specialized centers could improve survival and reduce comorbidities. Moreover, a standardized, team-based multidisciplinary approach within a regionalized care network could not only enhance patient outcomes but also facilitate pragmatic trial designs assessing both current and emerging therapies.

## 10. Conclusions

CS remains a condition with high morbidity and mortality due to its acute presentation, often posing a diagnostic challenge, and the rapid progression to multiorgan failure in some cases. These factors make CS a complex clinical scenario, highlighting the critical need to establish multidisciplinary protocols to guide interventions by involved healthcare professionals. This approach enables early and equitable access to specific treatments and hemodynamic support, aiming to improve patient prognosis.

## Figures and Tables

**Table 1:** Potential hemodynamic presentations of cardiogenic shock.

	Wet	Dry
Cold	Classic cardiogenic shock ↓ Cardiac index ↑ Systemic vascular resistance ↑ Pulmonary capillary wedge pressure	Euvolemic cardiogenic shock ↓ Cardiac index ↑ Systemic vascular resistance ↔ Pulmonary capillary wedge pressure
Warm	Vasodilatory cardiogenic shock ↓ Cardiac index ↓/↔ Systemic vascular resistance ↑ Pulmonary capillary wedge pressure	Vasodilatory shock (Not cardiogenic) ↑ Cardiac index ↓ Systemic vascular resistance ↓ Pulmonary capillary wedge pressure

**Table 2:** Bedside-focused echocardiograpy.

Initial Focused Echocardiographic Assessment of Cardiogenic Shock	Clinical Application of Initial Echocardiographic Data
▪ Assess left and right ventricular function and exclude the presence of intraventricular thrombus ▪ Ascertain the integrity of interventricular septum ▪ Evaluate aortic and mitral valve function ▪ Exclude significant pericardial effusion ▪ Assess hemodynamic profile: ○ Velocity–time integral (VTI) in left ventricular outflow tract (LVOT) correlates with stroke volume ○ Diastolic measurement of E/E’ waves can be obtained to estimate pulmonary wedge pressure ○ If tricuspid regurgitation is present, maximum jet velocity allows for estimating pulmonary pressure ○ Inferior vena cava (IVC) diameter can be used to estimate right atrial pressure	▪ Confirm shock has a cardiogenic origin ▪ Exclude mechanical complications, which would lead to specific treatment ▪ Predict prognosis. Low stroke volume estimated by VTI in LVOT is a strong marker of high risk associated with higher mortality ▪ Establish shock phenotype (left-sided/right-sided/biventricular). Depending on phenotype, different mechanical circulatory support systems might be appropriate. Intra-aortic balloon pumps (IABPs) and left ventricular microaxial flow pumps (MFPs) might be useful for left-predominant shock, while if severe right ventricular impairment is present a specific right-ventricular percutaneous device or veno-arterial ECMO could be necessary ▪ Exclude contraindications for specific MCS devices. Severe aortic regurgitation is a contraindication for IABP, MFP, and ECMO. Severe aortic stenosis or left ventricular thrombus contraindicates the insertion of MFP ▪ Adjust volume status. IVC dilatation and/or increased E/E’ values correlate with high filling pressures

**Table 3:** Inotropic and vasopressor drugs commonly used in clinical practice.

	Clinical Indication	Dosing	Major Side Effects
Dobutamine	Low CO (HF, cardiogenic shock, sepsis-induced myocardial dysfunction)	2–20 μg·kg^−1^·min^−1^ (max 40 μg·kg^−1^·min^−1^)	Tachycardia
			Ventricular arrhythmias
			Cardiac ischemia
			Hypertension (nonselective β-blocker patients)
			Hypotension
Norepinephrine	Shock (vasodilatory, cardiogenic)	0.01–3 μg·kg^−1^·min^−1^	Arrhythmias, bradycardia
			Peripheral ischemia
			Hypertension
Dopamine	Shock (cardiogenic, vasodilatory)	2.0–20 μg·kg^−1^·min^−1^ (max 50 μg·kg^−1^·min^−1^)	Hypertension (especially in nonselective β-blockers patients), ventricular arrhythmias, cardiac ischemia, tissue ischemia/gangrene (high doses or extravasation)
	HF		
	Symptomatic bradycardia unresponsive to atropine or pacing		
PDE-III inhibitors (milrinone, Amrinone, etc.)	Low CO (decompensated HF, after cardiotomy)	Bolus: 50 μg/kg (10–30 min) Infusion: 0.375–0.75 μg·kg^−1^·min^−1^ (dose adjustment in renal impairment)	Ventricular arrhythmias, hypotension
			Cardiac ischemia
			Torsade des pointes
			Caution in renal impairment
Levosimendan	Decompensated HF	Infusion: 0.05–0.2 μg·kg^−1^·min^−1^	Atrial tachycardia (especially if initial bolus), enhanced AV conduction, hypotension

CO, cardiac output; HF, heart failure; PDE-III, phosphodiesterase-III.

**Table 4:** Mechanical circulatory support devices.

	Impella CP Smart Assist	Impella 5.5 Smart Assist	Tandem Heart	IABP	Centrimag	VA-ECMO	Impella RP	Protek Duo
Flow	≤4.3 lpm C, Ax	≤6 lpm C, Ax	≤5 lpm C, Ce	0.5–1 lpm P	≤10 lpm C, Ce	≤10 lpm C, Ce	4–4.5 lpm C, Ce	4–5 lpm C, Ce
Implantation technique	Pe (S)	S	Pe (S)	Pe	S	Pe (S)	Pe	Pe (S)
Main access size	14 F (A)	21 F (A)	≥15 F (A)	7–8 F (A)	32 F inflow 22 F outflow	≥15 F (A)	23 F (V, femoral)	29, 31 F (V, jugular)
LV unloading	++	+++	+++	+	+++	-	NA	NA
CE mark	5 days	30 days	30 days	NA	30 days	30 days	14 days	30 days
Specific contraindications	Ao MV, LV T, AR *, AS *	Ao MV, LV T, AR *, AS *		AR *		AR *	RA/RV T	RA/RV T

* ≥ moderate. LV MCSs are depicted in red, LV or RV MCS in orange, biventricular MCS in green, and RV in blue. A: arterial, Ao: aortic, AR: aortic regurgitation, AS: aortic stenosis, Ax: axial, C: continuous, Ce: centrifugal, F: French, IABP: intra-aortic balloon pump, LV: left ventricular, MV: mechanical valve, NA: not apply, P: pulsatile, Pe: percutaneous, RA: right atrium, RV: right ventricle, S: surgical, T: thrombus, V: venous, VA-ECMO: veno-arterial ExtraCorporeal Membrane Oxygenation. -: no LV unloading; +: mild LV unloading; ++: moderate LV unloading; +++: large LV unloading.

**Table 5:** Patient selection criteria and contraindications in heart transplantation and LVAD.

	Selection Criteria	Contraindications
Heart transplantation	Advanced heart failure, refractory to current therapies, with an age limit set by the healthcare system (typically between 60 and 69 years), and without contraindications for cardiac surgery or immunosuppression	Ongoing infection (relative contraindication; may be an indication in infected LVAD) Severe peripheral or cerebral arterial disease Irreversible pulmonary hypertension with medical treatment Malignancy with poor prognosis Irreversible liver (cirrhosis) or kidney disease Multisystem systemic disease Other comorbidities with poor prognosis BMI > 35 before transplantation Ongoing excessive alcohol or drug use Psychological instability
Continuous-flow LVAD	Persistent severe symptoms despite optimal medical and device therapy, without severe right ventricular dysfunction or tricuspid regurgitation, who also present one of the following: LVEF < 25%, inability to exercise due to HF, or, with a peak O_2_ uptake < 12 mL/kg/min or < 50% of the predicted value Three or more hospitalizations for HF in the past year without an obvious precipitating cause Dependence on intravenous inotropic support or temporary mechanical circulatory support Progressive dysfunction of vital organs due to reduced perfusion and not excessively low ventricular filling pressures (PCWP ≥ 20 mmHg and SBP ≤ 90 mmHg or cardiac index ≤ 2 L/min/m^2^)	Significant right ventricular dysfunction or tricuspid regurgitation Inability to manage device due to psychosocial or psychiatric conditions Contraindication of long-term oral anticoagulation Active infection (to be individualized) Severe irreversible renal (or another organ) dysfunction Severe and incontrollable ventricular arrhythmias Technical inability to implant device

LVAD, left ventricular assist device; BMI, body mass index; LVEF, left ventricular ejection fraction; HF, heart failure; PCWP, pulmonary capillary wedge pressure; SBP, systolic blood pressure.
